# Comparative Analysis of the APOL1 Variants in the Genetic Landscape of Renal Carcinoma Cells

**DOI:** 10.3390/cancers14030733

**Published:** 2022-01-30

**Authors:** Maty Tzukerman, Yeela Shamai, Ifat Abramovich, Eyal Gottlieb, Sara Selig, Karl Skorecki

**Affiliations:** 1 Molecular Medicine Laboratory, Rambam Health Care Campus and Rappaport Faculty of Medicine, Technion, Haifa 31096, Israel; yeelashamai@gmail.com (Y.S.); seligs@technion.ac.il (S.S.); karl.skorecki@biu.ac.il (K.S.); 2 Rappaport Faculty of Medicine and Research Institute, Technion, Haifa 31096, Israel; ifat.a@technion.ac.il (I.A.); e.gottlieb@technion.ac.il (E.G.); 3 Department of Genetics and Developmental Biology, Rappaport Faculty of Medicine and Research Institute, Technion, Haifa 31096, Israel

**Keywords:** renal cell carcinoma, RCC tumorigenesis, CRISPR/cas9, *APOL1* gene, APOL1 risk variants, swollen cristae, mitochondria metabolism, Seahorse

## Abstract

**Simple Summary:**

Renal cell carcinoma (RCC) occurs at higher frequency in individuals of African ancestry, with well-recorded documentation in this community. This is most prominent in the context of chronic kidney disease. In turn, many forms of progressive chronic kidney disease are more common in populations of Sub-Saharan African ancestry. This disparity has been attributed to well-defined allelic variants and has risen in the parental populations to high frequency under evolutionary pressure. Mechanisms of increased kidney disease risk and cell injury, causally associated with these APOL1 gene variants, have been extensively studied. Most studies have compared the effects of ectopic overexpression of the parental non-risk APOL1 with the mutated risk variants in cellular and organismal platforms. In the current study, we have used CRISPR/Cas9 genetic engineering to knock out or modify the sequence of endogenous *APOL1* in RCC to mimic and examine the effects of these naturally occurring kidney disease risk allelic variants. Remarkably, these modifications to endogenous *APOL1* genes in RCC resulted in a set of prominent effects on mitochondrial integrity and metabolic pathways and disrupted tumorigenesis. These findings both clarify pathways of cell injury of APOL1 risk variants in cells of kidney origin and motivate further studies to examine the potential central role of APOL1 in the pathogenesis of renal cell carcinoma and its relation to chronic kidney disease in genotypically at-risk African ancestry individuals.

**Abstract:**

Although the relative risk of renal cell carcinoma associated with chronic kidney injury is particularly high among sub-Saharan African ancestry populations, it is unclear yet whether the *APOL1* gene risk variants (RV) for kidney disease additionally elevate this risk. APOL1 G1 and G2 RV contribute to increased risk for kidney disease in black populations, although the disease mechanism has still not been fully deciphered. While high expression levels of all three APOL1 allelic variants, G0 (the wild type allele), G1, and G2 are injurious to normal human cells, renal carcinoma cells (RCC) naturally tolerate inherent high expression levels of APOL1. We utilized CRISPR/Cas9 gene editing to generate isogenic RCC clones expressing APOL1 G1 or G2 risk variants on a similar genetic background, thus enabling a reliable comparison between the phenotypes elicited in RCC by each of the APOL1 variants. Here, we demonstrate that knocking in the G1 or G2 APOL1 alleles, or complete elimination of APOL1 expression, has major effects on proliferation capacity, mitochondrial morphology, cell metabolism, autophagy levels, and the tumorigenic potential of RCC cells. The most striking effect of the APOL1 RV effect was demonstrated in vivo by the complete abolishment of tumor growth in immunodeficient mice. Our findings suggest that, in contrast to the WT APOL1 variant, APOL1 RV are toxic for RCC cells and may act to suppress cancer cell growth. We conclude that the inherent expression of non-risk APOL1 G0 is required for RCC tumorigenicity. RCC cancer cells can hardly tolerate increased APOL1 risk variants expression levels as opposed to APOL1 G0.

## 1. Introduction

An increased relative risk of renal cell carcinoma associated with chronic kidney disease (CKD) is well documented among populations with sub-Saharan African ancestry, most notably African-Americans. The estimated proportion of renal cell carcinoma attributable to CKD within these populations accounts for 10%, while it is negligible among Caucasians. However, it is not known whether the APOL1 risk variants for CDK elevate the risk of renal cell carcinoma in these populations [1].

Apolipoprotein L1 (APOL1) is a circulating protein component of HDL (high-density lipoprotein) encoded by the APOL1 gene in humans and certain primate species [2,3,4]. In the kidney, APOL1 is expressed in podocytes and vascular components of the glomerulus, suggesting that it might play a role in renal diseases that affect the structure and function of these cells [2,5]. Indeed, APOL1 is associated with an increased risk of CKD, including end-stage kidney disease (ESKD), focal segmental glomerulosclerosis (FSGS), and HIV-associated nephropathy (HIVAN), specifically in individuals with sub–Saharan African ancestry [6,7,8]. The high CKD risk in this case is attributed to the high frequency of two APOL1 risk variants (RV) APOL1 G1 and APOL1 G2, which are associated with kidney disease, as opposed to the wild-type APOL1 G0 allele [6,9]. The high frequency of G1 and G2 alleles emanated from the protection they confer against African sleeping sickness caused by either *Trypanosoma brucei*, *Trypanosoma gambiense,* or *Trypanosoma rhodesiense* [10,11]. The expression of APOL1 is regulated in a cell-specific mode by specific protein 1 (Sp1) and interferon regulatory factors 1 and 2 (IRF1 and IRF2) [12]. Various roles were proposed for APOL1 proteins, including in autophagy [13,14,15], inflammatory cell death (pyroptosis) [15], impairment of vacuolar acidification [16], ER stress [17], lysosomal membrane permeability [18], mitochondrial dysfunction [19,20,21,22], and cationic channel activity at the plasma membrane [23,24]. To date, APOL1 function has been investigated either in cellular or organismal platforms that overexpress the APOL1 variants [15,16,20].

Attempts to fully elucidate the intricate mechanisms of APOL1-mediated renal injury to date have been inconclusive and sometimes contradictory. In part, this is attributable to the use of ectopic APOL1 variant constructs overexpressed in cell-culture platforms. In the current study, we utilize renal cell carcinoma (RCC) cells as a research model in which intrinsic high expression levels of APOL1 GO protein are tolerated and compatible with robust cell proliferation. Accordingly, to investigate the role of APOL1 and its risk alleles G1 and G2 in renal carcinoma cell growth, we set up tissue culture-based studies using the RCC 786-O cell line, a primary renal cell adenocarcinoma of a Caucasian male expressing APOL1-G0, thus circumventing the toxicity of ectopic overexpression. To further understand the specific contribution of the G1 and G2 APOL1 risk variants in kidney disease, we generated isogenic RCC clones expressing these variants. We took advantage of CRISPR/Cas9 genome editing to generate cells with levels of expression for APOL1 G1 and G2, comparable to those for G0, as well as APOL1 knock-out RCC. This methodology enables the expression of APOL1 G0, G1, and G2 variants on the same genetic background, avoiding the known modifying effects of varying haplotype backgrounds [25]. We demonstrate that the substitution of one nucleotide for expressing the APOL1 G1 or deletion of six nucleotides for expressing the APOL1 G2 led to a significant alteration in cell metabolism and tumorigenic potential in RCC cancer cells, as did deleting the endogenous gene.

## 2. Materials and Methods

### 2.1. Mice, Tumor Formation, and Tissue Handling

SCID/beige mice were purchased from Harlan Laboratories, Jerusalem, Israel. The mice were housed and maintained under specific pathogen-free conditions as instructed by the Committee for Oversight of Animal Experimentation at the Technion-Israel Institute of Technology, Haifa, Israel. The study was approved by the Institutional Animal Care Use Committee of the Technion (Protocol # IL-055-04-21). and 4 × 10^6^ cells were injected into the mices’ hind limb musculature. Mice were weighed and screened for tumor formation once a week. Mice were sacrificed using CO_2_ inhalation at 55 days following injection. Tumors were harvested, fixed in 10% neutral buffered formalin, transferred into 70% ethanol, and processed using a routine wax-embedding procedure for histological examination. Paraffin sections of six-micrometers were mounted on Super Frost Plus microscope slides (Menzel-Glaser, Braunschweig, Germany) and stained with hematoxylin/eosin.

### 2.2. Cell Lines and Tissue Culture

RCC 786-O (CRL-1932, ATCC) were grown at 37 °C in 5% CO_2_ in RPMI supplemented with 10% FCS, glutamine, and antibiotics. Human immortalized podocytes were grown under similar incubator conditions in DMEM supplemented with 10% FCS, glutamine, and antibiotics.

### 2.3. Antibodies

Anti-APOL1 (1:2500 HPA018885, Sigma, St. Louis, MO, USA); Anti-APOL1 5.17D12 (1 µg/mL, kindly provided by Genentech, San Francisco, CA, USA); Anti-APOL1 oligoclonal 3.7D6/3.1C1 (0.05 µg/mL, kindly provided by Genentech); Anti-Tubulin (1:5000 T5168, Sigma, St. Louis, MO, USA); Anti-LC3 (1:1000, PM036, MBL, Chicago, MA, USA); Anti-Calreticulin (1:1000, 12238, Cell Signaling, Danvers, MA, USA); anti-Cytochrome C (1:1000, sc-13156, Santa Cruz, Dallas, TX, USA); Anti-Calnexin (1:2000, ADI-SPA-860 Enzo, New York, NY, USA); Anti-SDHB (Succinate Dehydrogenase Complex Iron Sulfur Subunit B, 1:2500, LS C497529, LSbio); Anti-rabbit HRP (1:20,000; 111-035-144, Jackson ImmunoResearch, Baltimore, PN, USA); Anti-mouse HRP (1:10,000; 115-035-166, Jackson ImmunoResearch); Alexa Fluor 488 conjugated Donkey anti Rabbit (1:600; 711-545-152, Jackson ImmunoResearch).

### 2.4. Quantitative Real-Time RT-PCR

Total RNA was extracted from in vitro cultures, using the RNeasy^®^ Mini Kit (Qiagen, Germantown, MD, USA, 74104) and reverse transcribed with the 5X All-In-One RT MasterMix (Applied Biological Materials, Richmond, BC, Canada, G486) according to the manufacturer’s instructions. Quantitative real-time PCR was performed in triplicate, using the Applied Biosystems StepOnePlus Real-Time PCR system with Fast SYBR Green Master Mix (Applied Biosystems, Waltham, MA, USA, 4385612). All reactions were performed as follows: initial denaturation for 20 s at 95 °C, followed by 40 cycles of 95 °C for 15 s and 60 °C for 30 s. APOL1 gene-specific primer sequences for PCR were as follows: Fwd 5′-GCTGAACTGCCCAGGAATGA, Rev 5′-TTATCGTGCCAGTTTTTGTCTTTC. The relative expression levels of the target genes were compared with the endogenous control Hypoxanthine Phosphoribosyl transferase-1 (HPRT1) using the ΔΔCt method.

### 2.5. CRISPR/cas9 Methodology

CRISPR/cas9 mediated knock-out of APOL1: a guide RNA (gRNA) targeting a coding region in exon 5 of APOL1 (5′-TGAGGCCTGGAACGGATTCGTGG-3′), was designed using the Optimized CRISPR Design online tool (http://crispr.mit.edu, accessed on 13 December 2021) [26], and cloned into pSpCas9(BB)−2A-GFP (PX458), a gift from Feng Zhang (Addgene plasmid #48138) [26]. This plasmid was transfected into RCC cells using Lipofectamine™ 3000 Transfection Reagent (Invitrogen). GFP-positive single cells were collected into a 96-well plate using the FACS Melody cell sorter (BD Biosciences, San Jose, CA, USA) and expanded to obtain individual clones. Positive clones were identified by Western blot analysis to confirm the loss of APOL1 expression.

CRISPR/cas9 mediated homology-directed repair (HDR) for inserting the sequences of the G1 and G2 APOL1 alleles: For the generation of the APOL1 G1 variant: gRNA (5′ AGGAGTCAAGCTCACGGATGTGG-3′) and a ssODN template (5′-CTGGAAATGAGCAGAGGAGTCAAGCTCACTGATGTCGCCCCTGTAGGCTTCTTT-3′), were designed for an A to G single nucleotide substitution at position 1024 bp of the APOL1 coding sequence. For the generation of APOL1 G2 variant: sgRNA (5′-TAATTATAAGATTCTGCAGGCGG-3′) and ssODN (5′-AACATTCTCAACAATAAGATTCTTCAAGCAGACCAAGAACTGTGA-3′) were designed to generate an in-frame two amino acids deletion (Asn388 and Tyr389). gRNAs, cloned in the pSpCas9(BB)−2A-GFP (PX458) vector, were introduced together with the appropriate ssODN (100 µM) into RCC cells using Lipofectamine™ 3000 Transfection Reagent. GFP-positive single cells were collected as described above. Individual clones were screened by restriction fragment length polymorphism (RFLP). Briefly, genomic DNA was extracted from each clone and subjected to Polymerase chain reaction (PCR) using ApoL1 specific primers (Fwd 5-ACAAGCCCAAGCCCACGACC-3′ and Rev 5′-CCTGGCCCCTGCCAGGCATA-3′). The PCR reaction was as follows: 95 °C for 3 min; 35 cycles at 95 °C for 20 s, 63 °C for 20 s, and 72 °C for 30 s; 72 °C for 5 min. PCR products were digested with HindIII for identifying the G1 variant and with MlucI for identifying the G2 variant. The sequence of positive clones was confirmed by Sanger sequencing.

### 2.6. Western Blot

Cell proteins were extracted using extraction buffer: 150 mM NaCl, 50 mM Tris pH 8, 1 mM EDTA, 1% triton, and protease inhibitor cocktail (Sigma, St. Louis, MO, USA, P8340). Proteins (20–50 µg) were separated by SDS-PAGE on a 10–15% gel and blotted onto Western Bright NC Nitrocellulose Membranes (Amersham, St. Louis, MO, USA). The membranes were blocked with 5% nonfat dry milk (Santa Cruz sc-2325) and incubated with primary antibodies overnight at 4 °C. The membranes were then incubated with the appropriate secondary antibodies at room temperature for 1 h. The membranes were washed three times with TBST, and the proteins were visualized using chemiluminescence reagents (Advansta, San Jose, CA, USA, K-12045-D20). Fusion FX Spectra (VILBER) densitometry analysis was performed using TotalLab Quant software (TotalLab, Newcastle-Upon-Tyne, UK).

### 2.7. Immunofluorescence

For immunofluorescence, cells were seeded on fibronectin-coated coverslips in a 24 well plate. Cells were fixed in 4% paraformaldehyde, permeabilized with 0.15% TX-100 and Tween20 and incubated in a blocking solution (4% BSA, 0.15% Triton 0.15% Tween20). Cells were incubated with primary antibodies overnight at 4 °C, and then with a secondary antibody for 1 h at room temperature. Stained cells were mounted in Antifade Mounting Medium with DAPI (Vectashield H-1200) and visualized using a Confocal LSM 880 Upright Fluorescent Microscope (Zeiss, Berlin, Germany).

### 2.8. Fluorescent In Situ Hybridization (FISH)

RCC cells were treated with colcemid, harvested by trypsinization, treated with a hypotonic solution, and fixed with methanol/acetic acid (3:1). Cells were dropped on slides and hybridized by a standard FISH protocol to a probe generated from an APOL1-region BAC clone (RP1-6802). The probe was labeled with dUTP-digoxigenin. Hybridization was detected with anti-Dig-Rhodamine antibodies. DNA was stained with DAPI. Nuclei and chromosomes were visualized on a BX50 microscope (Olympus, Hamburg, Germany). Images were captured with an Olympus DP70 camera controlled by DP controller software (Olympus, Hamburg, Germany).

### 2.9. RCC Cell Fractionation

Cell fractionation was performed as described previously [27]. In brief, cells at 80–90% confluence were harvested by trypsinization. Cells were then manually homogenized in 10 mL of IB cells-1 buffer (225 mM mannitol, 75 mM sucrose, 30 mM Tris-HCl, 0.1 mM EGTA, pH 7.4) with about 100 strokes of a conical glass homogenizer, and sonicated (8 s × 3 times, wheel set to 3.5 in a Misonix Sonicator XL2020 Ultrasonic Liquid Processor sonicator). For separation of endoplasmic reticulum (ER), mitochondria, and mitochondria-associated membrane (MAM) fractions. Homogenized cells were first centrifuged at 600× *g* to yield total lysate extract. The total lysate was then centrifuged at 10,000× *g* to yield the crude mitochondria extract. Crude mitochondria fraction was separated from the MAM by fractionation on a 30% Percoll gradient (Sigma, P4937) at 95,000× *g*. The ER fraction was collected from the mitochondrial supernatant by centrifugation at 100,000× *g*. 10 µg of each fraction were loaded on a 4–20% Tris-Glycine gel. The various obtained protein fractions were immunoblotted into a nitrocellulose membrane and subjected to immune detection using the anti-APOL1 oligoclonal 3.7D6/3.1C1 specific rabbit monoclonal antibody. As a control, immune detection was also performed with various cell compartment-specific antibodies. These include: (1) Anti-Calreticulin that reacts with Calreticulin stored in the endoplasmic reticulum (ER); (2) Anti-Cytochrome C that reacts with Cytochrome C localized to the mitochondrial intermembrane space in mammalian cells; (3) Anti-SDHB that reacts with succinate dehydrogenase complex subunit B, iron sulfur Complex II of the respiratory chain, which is specifically localized to the mitochondrial inner membrane; (4) Anti-Tubulin that reacts with Tubulin localized to the cytoplasm; (5) Anti-Calnexin that reacts with Calnexin localized to the endoplasmic reticulum.

### 2.10. Cell Proliferation

Cells were cultured in a 96 well plate with a regular medium (5000 cells per well). At 24 h, the growing medium was replaced with a medium containing glucose or galactose, and the plates were placed in the Incucyte zoom 2016B system (Essen Bioscience, Ann Arbor, MI, USA). Cells images were captured every three hours for three days. Proliferation analysis was performed using the Incucyte Zoom software. Alternatively, cell proliferation was analyzed using the Cell Titerglow (Promega, Fitchburg, WI, USA) according to the manufacturer’s instructions.

### 2.11. Autophagy Flux Measurements

Cells (1.5 × 10^6^ cells/10 cm dish) were grown in a regular medium or Earle’s Balanced Salt Solution, EBSS (Biological Industries, Bet-Haemek, Israel) for starvation conditions, with or without Bafilomycin A1 (Santa Cruz, sc-201550A). For Western blot analysis, cells were harvested at 0, 3, 6, and 9 h following starvation for protein extraction. Protein samples were separated on 15% SDS-PAGE gel, transferred to a membrane, and LC3 protein was detected using anti LC3 antibodies. For the Tandem Sensor RFP-GFP-LC3B methodology, the Premo™ Autophagy Tandem Sensor RFP-GFP-LC3B Kit (Invitrogene, Carlsbad, CA, USA, P36239) was used according to manufacturer instructions. In brief, cells grown in regular medium or under starvation conditions, with or without Bafilomycin A1, were transduced with the BacMam 2.0 RFP-GFP-LC3B reagent and visualized using standard GFP (green fluorescent protein) and RFP (red fluorescent protein) settings of the Confocal LSM 880 Upright Fluorescent Microscope (Zeiss, Berlin, Germany). For image-based analyses of autophagy, red and green dots were quantified using the Imaris Image Analysis Software, and the ratio of green dots to red dots was determined.

### 2.12. Bioenergetic Measurements

Oxygen consumption rate (OCR) and extracellular acidification rate (ECAR) were measured using the Seahorse XFe96 analyzer (Agilent, Santa Clara, CA, USA). Cells were seeded into Seahorse XFe96 plates at 2 × 10^4^ cells/well 24 h prior to measurements. The following day, the medium was replaced with 180 μL of unbuffered assay media (Sigma, St. Louis, MO, USA, D5030) supplemented with 5 mM glucose (Sigma, G8270,)or 5 mM galactose (Sigma, G0625), 1 mM pyruvate (Sigma, P8754), and 2 mM Glutamine (pH 7.4) (Sigma, G3126). Cells were then incubated at 37 °C in a non-CO_2_-incubator for 45 min. During the steady state, the basal OCR and basal ECAR were determined. Then, respiratory and glycolytic rates were measured in response to sequential injections of 2 µM oligomycin (Complex V inhibitor) (Sigma, 75351), 1.0 μM carbonyl cyanide-4-(trifluoromethoxy)-phenylhydrazone (FCCP, an uncoupling agent of mitochondrial respiration to achieve the maximal respiration rate) (Sigma, C2920), and 50 μM rotenone and antimycin A mixture (Complex I/III inhibitor) (Sigma, R8875 and A8674). Cells were immediately lysed using 30 μL/well Lysis Reagent A (Bio-Rad, Hercules, CA, USA), and proteins were quantified using the Laury protein assay kit (Sigma, 47641). Glycolytic rates in individual wells were normalized to milligrams of total protein, as quantified by a standard curve. Two independent experiments were performed, and all data were presented as M ± S.D.

### 2.13. Retroviral Infections

APOL1 gene variants G0, G1, and G2 cDNAs were cloned into pBABE-puromycin retroviral vectors using the restriction sites of EcoRI and BamHI. To generate retroviral particles, the viral packaging cell line GP2- 293 (stably expressing the gag and pol genes) was cotransfected with the pBABE constructs and a plasmid containing the vesicular stomatitis virus glycoprotein gene (VSVG). Transfection was performed using the Lipofectamine™ 3000 Reagent (Invitrogen, Carlsbad, CA, USA, L3000-008). 48 h post-transfection, the supernatant containing the viruses was collected and filtered through a 0.45-μm PVDF filter. Viruses were used to infect the RCC null cells in the presence of 6 µg/mL polybrene (Millipore, TR-1003-G).

## 3. Results

### 3.1. APOL1 Expression in RCC 786-O

**Figure 1:**
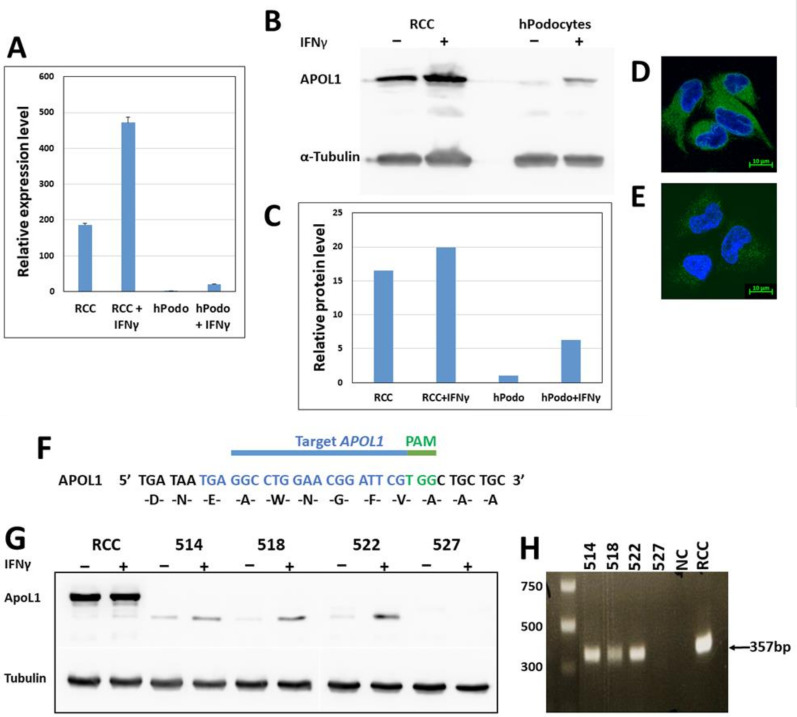
APOL1 expression in RCC 786-O prior and post gene knock-out. (**A**) APOL1 mRNA levels as determined by RT-qPCR analysis. APOL1 transcription was determined in RCC and normal immortalized podocytes (hPodo), with and without treatment with interferon gamma (IFNγ. mRNA expression values are presented as mean ± SE; (**B**,**C**) APOL1 expression at the protein level as determined by Western blot analysis with the anti-APOL1 HPA 018885, with and without treatment with IFNγ; (**D**,**E**) Expression of APOL1 in RCC (**D**) and podocytes (**E**) was examined using immunofluorescence analysis with anti-APOL1 antibodies (anti APOL1 HPA 018885) (green). Nuclei were stained with DAPI (blue). Bar = 10 µm; (**F**) Knock-out of *APOL1* in RCC cells was performed using CRISPR/Cas9 mediated genome editing, targeting a region in exon 5 of the APOL1 variant B1 (transcript ENST00000319136.8). The guide RNA (gRNA) target (nucleotide 267-302) and PAM sequences TGG are depicted in blue and green, respectively; (**G**) Analysis of APOL1 expression at the protein level by Western blot analysis of single cell derived clones obtained following gene editing; (**H**) PCR analysis of genomic DNA of single cell derived clones obtained following gene editing. DNA was amplified using exon 5 specific primers (described in the Section 2). The uncropped Western blots are shown in Figure S3.

We first determined APOL1 copy number and allelic variants in RCC 786-O cells. FISH analysis on RCC nuclei and metaphase chromosomes indicated three copies of *APOL1* in these cells (Figure S1). Sequence analysis demonstrated that all APOL1 copies are the WT G0 variant embedded in the haplotype background of E150, I228, K225 [25] (data not shown). qPCR analysis of APOL1 in RCC 786-O demonstrated a ~200 fold higher expression level than unstimulated immortalized normal podocytes. Interferon Gamma (IFNγ), a strong regulator of *APOL1* [12], elevated APOL1 expression both at the mRNA and protein levels in RCC (Figure 1A–C). Immunofluorescence (IF) analysis confirmed high protein levels of APOL1 in RCC compared with immortalized normal podocytes (Figure 1D,E).

### 3.2. Knock out of APOL1 in RCC 786-O Cells

To investigate the impact of APOL1 in RCC cells, we first eliminated its expression by non-homologous end joining (NHEJ) implemented by CRISPR/Cas9 editing. For this purpose, we utilized a guide RNA (gRNA) that targets a region in APOL1 exon 5 (Figure 1F). This procedure yielded four clones in which APOL1 expression was abolished, based on western blot analysis (Figure 1G). PCR analysis using primers that delimit APOL1 exon 5 (8418 bp–8764 bp) yielded a 346 bp DNA fragment in three of these clones (514, 518, and 522) (Figure 1H). Sequence analysis of these PCR products demonstrated a scrambled sequence. Clone 527, which did not yield any PCR product or APOL1 protein, was chosen to represent APOL1-deficient RCC cells (RCC null).

### 3.3. Genome Editing of APOL1 to the G1 and G2 RV in RCC Cells

**Figure 2:**
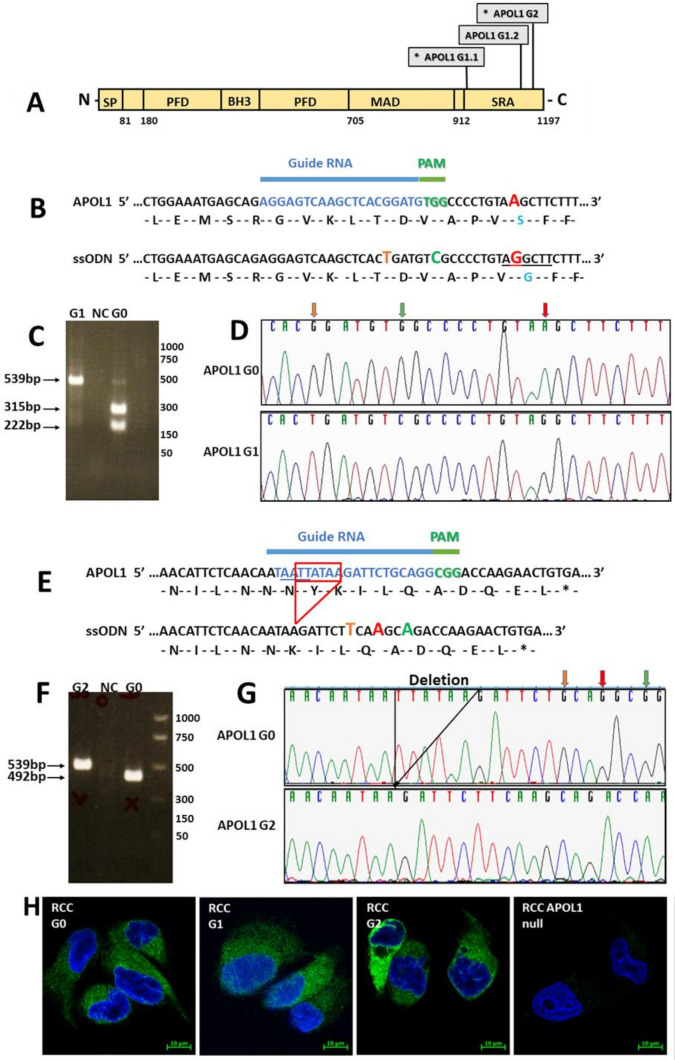
CRISPR/Cas9-mediated generation of APOL1 risk variants. (**A**) The positions of G1 and G2 polymorphisms in the human APOL1 protein. APOL1 consists of a cleavable N-terminal signal peptide (SP), a pore-forming domain (PFD), a membrane-addressing domain (MAD), and a serum resistance-associated (SRA)-interacting domain. The G1 and G2 renal risk polymorphisms are located near the C-terminus of the protein, at the. SRA-interacting domain; (**B**) gRNA (blue) and PAM sequence TGG (green) used for knocking in the G1 variant (positioned in exon 7 of APOL1). The single-stranded oligodeoxynucleotides (ssODN) used for homology-directed repair (HDR) appear below the targeted region. The ssODN was designed to alter three nucleotides: an A to G nucleotide change as indicated in red, to insert the G1 variation, and two synonymous changes to prevent re-annealing of the gRNA following HDR: a G to C change in the PAM sequence (green) and a G to T change in the region bound by the gRNA (orange). The A to G change abolishes a HindIII restriction site (underlined). A positive clone of APOL1 G1 knock-in was validated by PCR with specific primers (described in the Materials and Methods section) following digestion with HindIII restriction enzyme (**C**) and by Sanger sequencing (**D**). Orange, green, and red vertical arrows in (**D**) depict the positions of the edited nucleotides; (**E**) gRNA (blue) and PAM sequence CGG (green) used for knocking in the G2 variant. The single stranded oligodeoxynucleotide (ssODN) used for homology-directed repair (HDR) appear below the targeted region. The ssODN was designed to delete 6 nucleotides (TTATAA), depicted by the red frame, generating the G2 variant. In addition, the ssODN was designed to incorporate three synonymous changes: a G to T (orange) and G to A (red) changes in the target regions of the gRNA, and a G to A change in the PAM sequence, to prevent re-annealing of the gRNA (* = stop codon). The 6-bp deletion abolishes a MlucI restriction site (AATT, underlines in the target region). An APOL1 G2 positive clone was confirmed by PCR specific primers (described in the Materials and Methods section) following digestion with MlucI (**F**) and by Sanger sequencing (**G**). Orange, green, and red vertical arrows in (**G**) depict the positions of the edited nucleotides. (**H**) Expression of APOL1 G0, G1, and G2 in RCC cells was examined using immunofluorescence analysis with anti-APOL1 antibodies (anti APOL1 5.17D12) (green). Nuclei were stained with DAPI (blue), Bar = 10 µm.

To eliminate the influence of diverse genetic backgrounds, we generated isogenic RCC cells expressing the G1 and G2 RV using CRISPR/Cas9 homology-directed repair (HDR). G1 is a two coding variant comprising haplotype, S342G and I384M, whereas the G2 contains a 6-bp deletion (del.N388/Y389) (Figure 2A) [6,9]. Since it has been shown that it is the S342G variant of G1 that is responsible for kidney cell injury by a single mutation in the APOL1 coding region [16], we used a gRNA and a single-stranded oligo donor (ssODN) corresponding to nucleotides 979-1072 (Figure 2B). Screening of single cell-derived populations (720 clones) by restriction fragment length polymorphism (RFLP) analysis yielded a single clone in which all three alleles of APOL1 were edited to carry the G1 variant, a finding also verified by Sanger sequence analysis (Figure 2C,D).

For generating the G2 variant in RCC cells, we utilized a gRNA and an ssODN corresponding to nucleotides 1146–1197 bp of the APOL1 coding region (Figure 2E). This ssODN included deletion of 6 bp corresponding to amino acids N388 and Y389. Screening of 700 single cell-derived cell populations using RFLP yielded one clone in which the 6-bp deletion occurred in all three alleles (Figure 2F,G). Immunofluorescence analysis using an anti-APOL1 antibody exhibited similar expression levels of APOL1 G0, G1, and G2 in RCC cells (Figure 2H). Using the CRISPR methodology, we identified only one single cell derived population of RCC G1 and RCC G2, although we screened hundreds of clones. Nevertheless, a careful examination taken at the genomic DNA, mRNA expression, and protein levels indicated that this methodology specifically edited the APOL1 gene in RCC cells.

Taken together, via gene editing, we generated three RCC cell lines with the same genetic background, endogenously expressing either the G0, G1, or G2 APOL1 variants, thus enabling comparative analysis of the effects of each variant in RCC cells.

### 3.4. The Effect of APOL1 G0, G1, and G2 on Growth and Proliferation of RCC Cells

**Figure 3:**
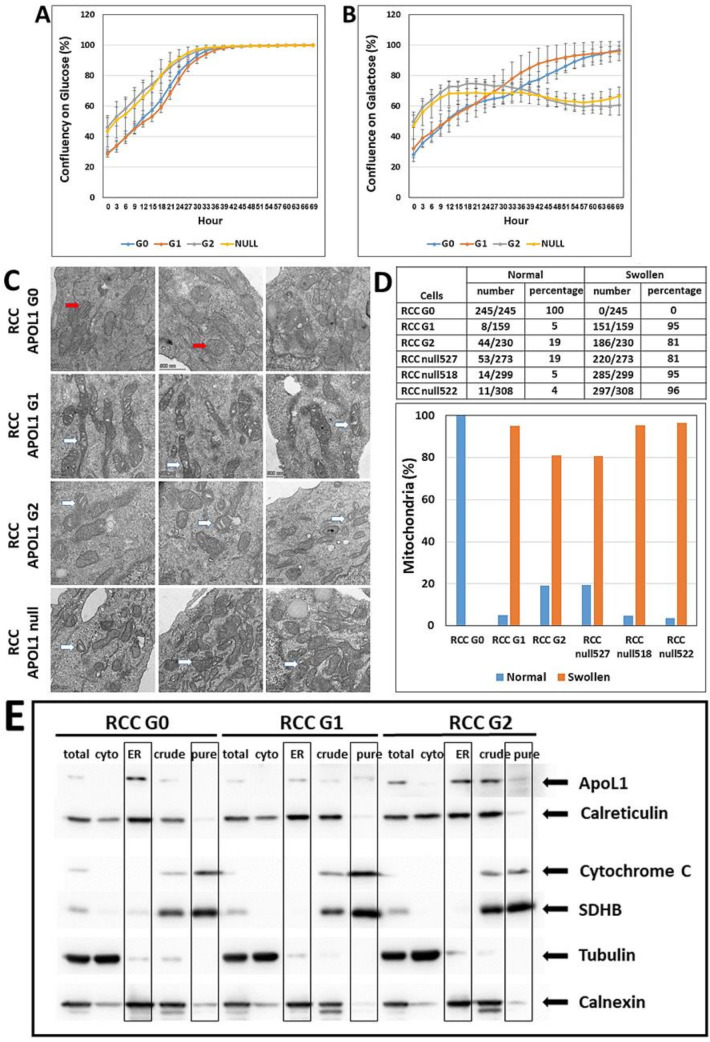
The effect of APOL1 editing on proliferation and mitochondrial shape. APOL1 G0, G1, G2, and null RCC cells were grown in a culture medium containing low concentrations of either glucose or galactose (5 mM each). The proliferation rate was measured using the Incucyte Live-Cell Analysis System at three hour intervals for three days. (**A**,**B**) Cell confluency at each time point, indicating the proliferation rate in the presence of glucose or galactose. Values of cell confluency are expressed as mean ± SE; (**C**) Mitochondrial shape in G0, G1, G2, and null RCC cells were examined using the Talos L120C Transmission Electron Microscope (TEM) (Waltham, MA, USA), at 120 kV (Bar = 500 nm). Red and white arrows indicate clear or swollen mitochondrial cristae, respectively; (**D**) TEM pictures were screened for mitochondria with swollen cristae appearance versus mitochondria with normal clear cristae appearance; (**E**) Cellular localization of APOL1 in RCC G0, RCC G1, and RCC G2 cells was examined using differential centrifugation analysis. Protein fractions were collected as follows: Total protein (total), cytoplasmic fraction (cyto), endoplasmic reticulum fraction (ER), crude mitochondrial fraction (crude), and pure mitochondrial fraction (pure). Protein fractions (10 µg) were separated on SDS-PAGE gel, immunoblotted, and subjected to immune-detection using an anti-APOL1 specific antibody. Antibodies used for control: anti-Calreticulin for endoplasmic reticulum (ER), anti-Cytochrom C for the mitochondrial intermembrane space, anti-SDHB for mitochondrial inner membrane, anti-Tubulin for the cytoplasm, anti-Calnexin for the endoplasmic reticulum (ER). The uncropped Western blots are shown in Figure S4.

Based on differences observed in the growth rate of RCC G0, G1 and G2, and RCC null cells, we hypothesized that the APOL1 variants might influence mitochondrial function in RCC cells, which in turn could affect cell proliferation rate. Mitochondrial dysfunction is difficult to study in a high glucose medium as rapidly proliferating cancer cells are highly glycolytic in such medium [28]. However, the substitution of glucose for galactose in the growth medium enables reliable measurements of proliferation rate and mitochondrial metabolism [29,30]. Both luminescent cell viability assays and live cell analysis demonstrated comparable proliferation rates for RCC G0, G1, and G2 and for RCC null cells when grown on glucose-containing medium (Figure S2 and Figure 3A). However, the proliferation rate of RCC APOL1-null cells was significantly reduced when grown on a galactose-supplemented medium, as compared with parental RCC cells (G0) (Figure 3B), suggesting that the loss of APOL1 impedes the growth of RCC cells on galactose as a carbohydrate source of energy. Interestingly, the proliferation rates of RCC G0 and G1 are similar, while RCC G2 grows at similar rates as the APOL1-null line, as demonstrated by the ratio of proliferation rate on glucose to galactose for the various RCC cells (Figure S2B–D and Figure 3B). These results indicate that, at least in respect to the utilization of different carbohydrate energy sources, the APOL1 G1 and G2 variants clones behave differently.

### 3.5. Phenotypic Appearance of the Mitochondria in RCC G0, RCC G1, and RCC G2 and in RCC Null Cells

One of the major questions regarding APOL1 nephropathy is the mechanism whereby APOL1 RV elicit their toxicity in cells [31]. Among other hypotheses, it has been suggested that APOL1 RV induce cell death via translocation and opening of the mitochondrial permeability transition pores [32]. Mitochondrial dysfunction was demonstrated to involve self-aggregation of the APOL1 G1 and G2 RV following transport into the mitochondria [32]. Therefore, we compared the mitochondrial ultrastructural morphology in RCC G0, G1, G2, and null RCC cells by Transmission Electron Microscopy (TEM). Indeed, striking differences in mitochondrial morphology were observed among the various RCC- expressing different APOL1 variants (Figure 3C). In RCC G0 elongated mitochondria were observed with clear mitochondrial cristae. In RCC G1, we similarly observed elongated mitochondria; however, many of the mitochondria were characterized by numerous swollen cristae. On the other hand, in RCC G2 we observed shorter mitochondria with many swollen cristae. In RCC null cells, we could observe various lengths of mitochondria with many swollen cristae (Figure 3C). Counting the number of mitochondria with swollen cristae versus normal cristae revealed striking differences between the parental RCC G0 to the G1 and G2 RV RCC cells, as well as to three different clones of RCC null cells (Figure 3D). Thus, while mitochondria length remains normal in RCC G1 as opposed to RCC G2, both G1 and G2 RV demonstrate a swelling of mitochondrial cristae, suggesting that the APOL1 RV severely disrupt mitochondrial function in these cells. Similarly, severe mitochondrial cristae swelling is observed in APOL1 null RCC cells.

### 3.6. Cellular Localization of APOL1 in RCC G0, G1, G2, and Null Cells

A previous publication reported translocation of APOL1 isoforms into the mitochondrial matrix where APOL1 RV form higher-ordered oligomers [32]. To examine the cellular localization of the various APOL1 variants in RCC cells, we performed fractionation by differential centrifugation, as previously described [27]. This process yielded the following protein fractions: total protein, cytoplasmic fraction (Cyto), endoplasmic reticulum (ER) fraction, crude mitochondrial fraction, and pure mitochondrial fraction (ER). Analysis of the various protein fractions with multiple antibodies indicated that APOL1 G0, G1, and G2 are mainly localized in the ER, and to a minor extent in the total protein fraction (Figure 3E). APOL1 G2 is also detected in the crude and, to a lesser extent, in the pure mitochondrial fractions. However, this could result from minor contamination of the pure mitochondrial fraction with the ER fraction, as demonstrated by the immunoreaction of Calnexin in the mitochondria fractions, which normally is absent. Taken together, this analysis demonstrates the localization of the APOL1 G0, G1, and G2 in the ER in RCC cells and their absence from the mitochondria.

### 3.7. The Effect of APOL1 Variants on Mitochondrial Metabolism in RCC Cells

**Figure 4:**
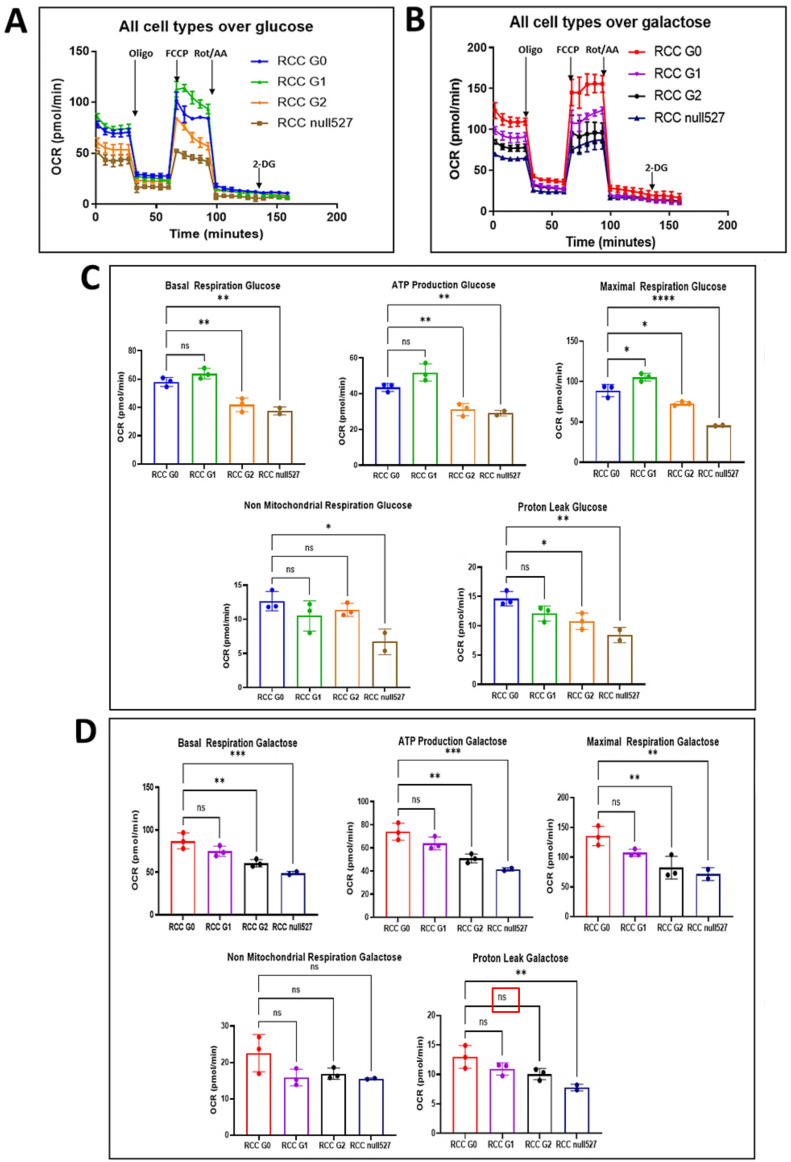
The effect of APOL1 editing on mitochondrial metabolism. (**A**,**B**) Oxygen consumption rate (OCR) via oxidative phosphorylation (OXPHOS) was measured in APOL1 G0, G1, G2, and null RCC cells grown in either glucose (**A**) or galactose (**B**). Cells were subjected to a mitochondrial stress test prior to real-time measurements of OCR using the Seahorse XFe96 extracellular flux analyzer. Serial injections of metabolic inhibitors, Oligomycin (Oligo), FCCP, and a combination of Rotenone and Antimycin A (Rot/AA), complex I/III inhibitor, was performed at the time points depicted in the graph by vertical arrows. (**C**,**D**) Calculated values of basal respiration, ATP production, maximal respiration, non-mitochondrial respiration, and proton leak for the various RCC cells grown in glucose (**C**) or galactose (**D**). The analysis was repeated 3 times. Basal respiration rate, ATP production, maximum respiration rate, non-mitochondrial respiration, and proton leak are expressed as mean ± SE. (* *p* < 0.05, ** *p* < 0.01, *** *p* < 0.001).

Alteration of mitochondrial morphology can reflect altered mitochondrial function [33]. As dysfunctional mitochondria have been shown to contribute to tumor progression [34], we examined whether the metabolism and the bioenergetics are altered in RCC cells that express the APOL1 RV and manifest the swelling morphology. During tumorigenesis, energy metabolism can be mediated through mitochondrial oxidative phosphorylation (OXPHOS) or via glycolysis, and their levels can be analyzed respectively by measuring the oxygen consumption rate (OCR) and extracellular acidification rate (ECAR). To investigate whether the APOL1 variants affect OCR or ECAR in RCC cells grown on glucose or galactose, we performed real-time measurements of OCR and ECAR during mitochondrial and glycolysis stress tests, using the Seahorse XFe96 extracellular flux analyzer (Figure 4A,B). Basal measurements of OCR values when grown on glucose-supplemented medium indicated higher levels of basal respiration in RCC G0 parental cells and RCC G1 cells (~60–65), compared to RCC null and RCC G2 (~35–40). When grown on a galactose-supplemented medium, RCC G0 and RCC G1 similarly demonstrated higher levels (~70–80) compared with RCC null and RCC G2 (~50–60) (Figure 4C,D). Injection of Oligomycin inhibits ATP synthase, thereby decreasing electron flow through the mitochondrial electron transport chain (ETC) and reducing the values of mitochondrial respiration or OCR, a decrease linked to cellular ATP production. ATP production measurements in glucose-containing media following an injection of Oligomycin yielded values of ~42–50 for RCC G0 and RCC G1, as compared with ~29–30 for RCC null and RCC G2, and ~60–70 and ~40–50 for galactose containing media, respectively (Figure 4C,D). Measurements of the maximal respiration following injection of the uncoupler fluoro-carbonyl cyanide phenylhydrazone (FCCP) disrupt the mitochondrial proton gradient and the mitochondrial membrane potential; hence, the electron flow through the ETC is uninterrupted, and the OCR reaches its maximal rate. This enables calculating the spare respiratory capacity, the difference between the basal and maximal OCR. The results we obtained indicated that the spare respiratory capacity is higher in RCC G0 and RCC G1 compared to RCC null and RCC G2 when grown on glucose (G0, G1: ~80–110, G2 and null: ~40–70), as well as on galactose (G0, G1: ~100–130, G2 and null: ~40–50) (Figure 4C,D). Interestingly, when grown on glucose, RCC G1 cells exhibited somewhat higher OCR values than RCC G0. However, in galactose-containing media, when cells rely heavily on OXPHOS for energy production, a reversal was observed, and RCC G0 OCR values were higher than those of RCC G1, G2, and APOL1 null. This indicates that substrate availability for energy metabolism might play an important role in APOL1 RV function in RCC cells. OCR values dramatically decreased following injection of Oligomycin due to the inhibition of the proton flux through ATP synthase. Hence the change between OCR at basal levels and after oligomycin injection indicates the fraction of oxygen being consumed for ATP production, and with that, the extent of OXPHOS in these cells. In addition, we tested the effect of a combination of rotenone (a complex I inhibitor) and antimycin A (a complex III inhibitor). This combination shuts down mitochondrial respiration and reveals the respiration values stemming from non-mitochondrial metabolism. These assays indicate that regarding APOL1 variants in RCC cells, RCC G0 exhibits the highest values of ATP-coupled respiration. Of note, the Glycolysis Stress test that measures the capacity of glycolysis pathways following glucose starvation did not yield any significant differences between the different RCC cell lines.

Together, these results suggest that parental RCC cancer cells, expressing the APOL1 G0 variant, respond better to environmental changes by adjusting their energy production through mitochondrial respiration, as opposed to RCC cells expressing APOL1 RV or RCC APOL1 null cells.

### 3.8. Re-Expression of Exogenous APOL1 G0, G1, and G2 cDNAs in RCC Null Cells

The forgoing altered proliferation rates and mitochondrial phenotypes in RCC null, G1, and G2 cells were obtained in clones selected after CRISPR mediated genome editing. To verify that these phenotypes are directly related to the knock-out of the APOL1 gene or the knock-in of the G1 and G2 variant sequences, we first sequenced the full-length APOL1 cDNAs derived from the RCC parental cells, and from the RCC cells isolated following genome editing. The results confirmed the correct sequence of APOL1 G0, G1, and G2, with no additional sequence alternation at unrelated positions of the cDNA.

**Figure 5:**
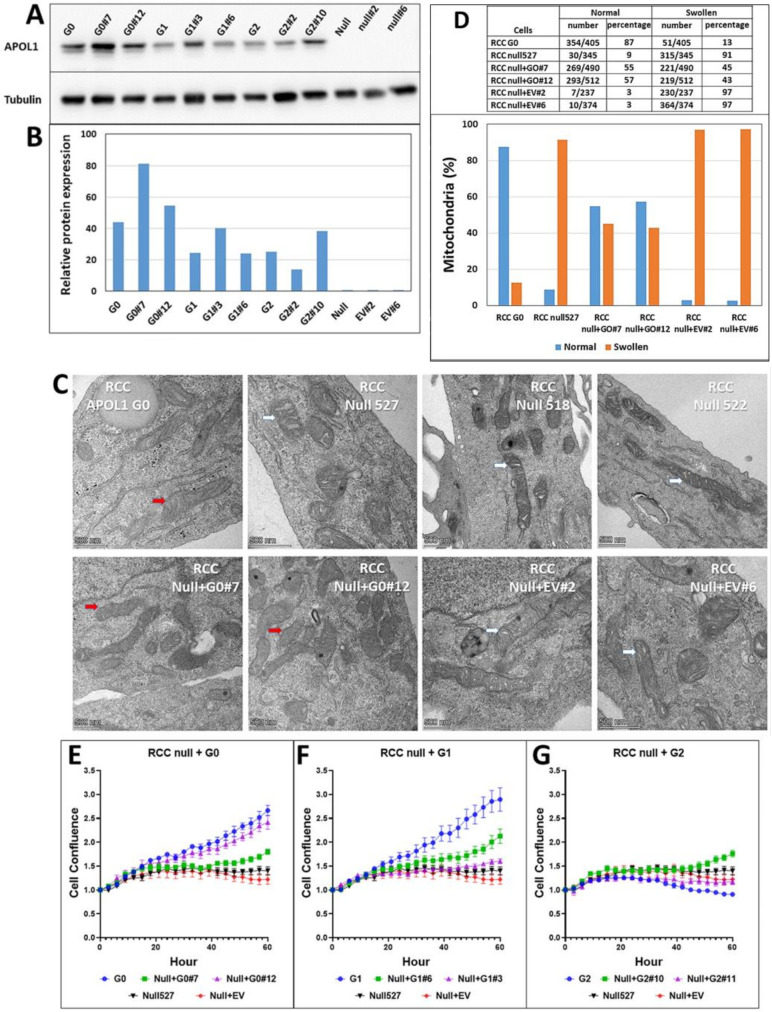
Ectopic expression of APOL1 G0, G1, and G2 in APOL1-null RCC cells. APOL1 G0, G1, and G2 cDNAs were ectopically expressed in APOL1-null RCC null cells. Cells transduced with a pBABE empty vector (EV) were used as control. (**A**) Western blot analysis of APOL1 protein levels in RCC G0, RCC G1, RCC G2, and RCC null, and from two puromycin resistant single cell derived populations of RCC null cells transduced with each of the APOL1 G0, G1, G2 cDNAs, or with an EV, as indicated. An anti-α-Tubulin antibody was used as a protein loading control; (**B**) Quantification of protein expression levels in each cell population; (**C**) Mitochondrial shape of the variously described clones was examined by Transmission Electron Microscope (TEM) (Waltham, MA, USA), and red and white arrows indicate clear or swollen mitochondrial cristae, respectively (Bar = 500 nm); (**D**) Mitochondria with normal and swollen cristae were counted; the percentage of normal cristae (Normal) and swollen cristae (Swollen) in RCC G0, RCC null, and two single cell-derived populations of RCC null that exogenously express the APOL1 G0 cDNA or an empty plasmid are presented in the upper table and lower graph; (**E**–**G**) Proliferation capacity of RCC null cells ectopically expressing APOL1 G0 (**E**), G1 (**F**), or G2 (**G**), as determined by the Incucyte Live-Cell Analysis System (Incucyte zoom 2016B). Two single cell-derived populations of RCC null + G0, RCC null + G1, and RCC null + G2 were grown in a Galactose-containing medium and examined for their proliferation capacity. RCC null cells and RCC null + empty vector (EV) were used for control and are presented in (**E**–**G**) as the baseline. Normalized values of cell confluence are expressed as mean ± SE. The uncropped Western blots are shown in Figure S5.

We next transduced exogenous copies of either APOL1 G0, G1, or G2 cDNA into RCC null cells and isolated antibiotic-resistant, single-cell derived populations expressing each of the APOL1 variant proteins. The expression of the exogenous APOL1 protein in these clones was validated by Western blot analysis (Figure 5A,B). Control cells transduced with an empty plasmid did not demonstrate any APOL1 protein, similar to the parental RCC null cells.

Next, we examined whether ectopic expression of APOL1 G0 in RCC null cells rescues the swollen mitochondrial cristae phenotype. TEM analysis of two single cell-derived populations (clone #7 and clone #12) of RCC null+ APOL1 G0 indicated that many of the cristae had restored normal morphology (Figure 5C,D). Altogether, in more than 57% of the mitochondria in these cells, clear cristae were observed (Figure 5D). On the other hand, introducing the empty vector did not rescue the aberrant mitochondria phenotypic appearance (Figure 5D). The mitochondrial phenotype of RCC null cells expressing the APOL1 G1 or G2 RV were not examined, being uninformative since they themselves have swollen cristae.

Taken together, our findings indicate that the aberrant phenotypic appearance of swollen cristae in the mitochondria of RCC APOL1-knock-out cells are a direct consequence of APOL1 loss of function.

### 3.9. Re-Expression of APOL1 G0, G1, or G2 cDNAs in RCC Null Cells Influences Cell Growth Capacity

RCC parental cells, RCC null cells, and RCC cells that express endogenous APOL1 RV G1 or G2 differ in their proliferation rates when grown in galactose-containing media (Figure 3A,B). To examine whether re-expression of the APOL1 G0, G1, and G2 rescues the reduced proliferation rate of RCC null cells, we used Incucyte Live-Cell Analysis. For this purpose, two single cell derived clones of RCC null + G0, RCC null + G1, or RCC null + G2 were seeded in media containing either glucose or galactose and examined for their proliferation capacity in comparison to RCC G0 parental cells and RCC null cells. Growing on glucose, the various clones exhibited a similar proliferation capacity. However, when growing on galactose, re-expression of APOL1 G0 in RCC null cells reversed the attenuated proliferation rate observed in APOL1 null RCC cells (Figure 5E). In RCC null cells + APOL1 G1, the proliferation rate rose to the level observed for RCC G1 in clone six; however, clone three showed only minor rescue, probably due to clonal variation (Figure 5F). Expression of APOL1 G2 in RCC null cells did not improve the proliferation rate relative to RCC G2 (Figure 5G). As expected, transfection of the empty vector (EV) did not influence the proliferation rate. Thus, similar to the mitochondrial phenotypes, the differences in proliferation rate between RCC G0, G1, G2, and RCC null can be specifically related to the changes induced by editing the APOL1 gene in the parental RCC genomic landscape. These results further support the conclusion that phenotypes associated with APOL1 G1, G2, and null alleles are induced by the loss of function of APOL1 activity.

### 3.10. APOL1 G0 Inhibits Autophagy in RCC Cells

**Figure 6:**
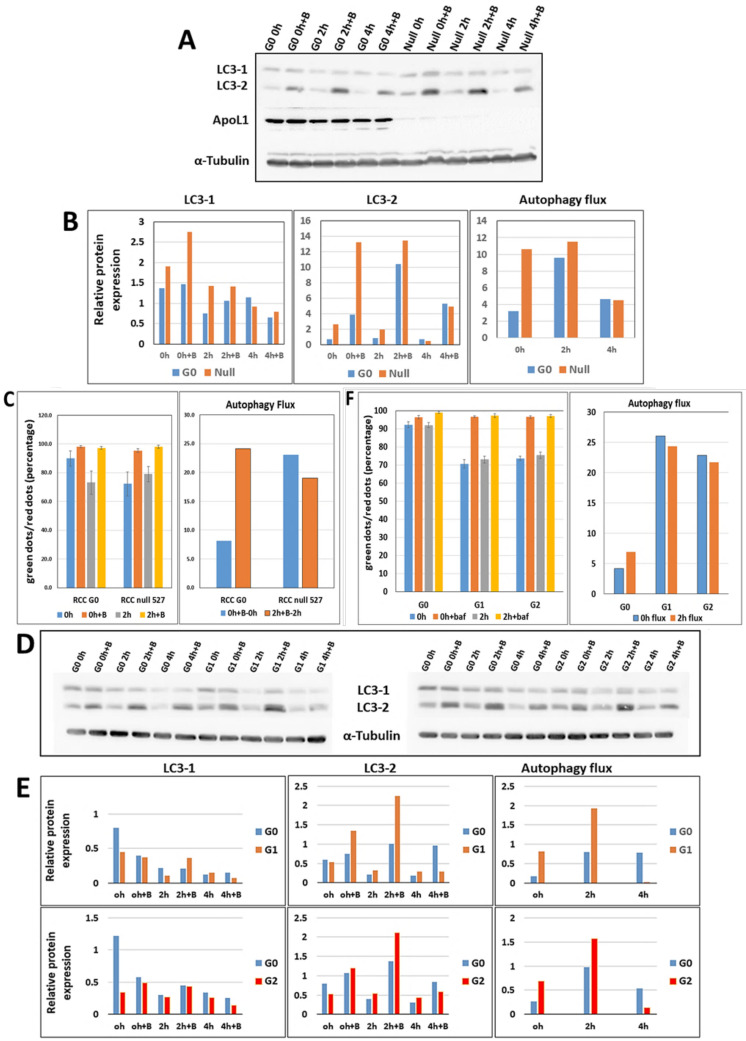
Autophagy flux in APOL1 G0, G1, G2, and null RCC cells. (**A**) Proteins were extracted from RCC G0 and RCC null cells grown in normal medium (0 h) and under starvation conditions for two and four hours, without or with Bafilomycin A1 inhibitor. The protein levels of LC3-I and LC3-II were determined by Western analysis using the anti-LC3 antibody. Anti-APOL1 antibody was used to confirm the absence of APOL1 expression in RCC null cells, and anti-αTubulin was used as a protein loading control. Protein levels were quantified as described; (**B**) Autophagy flux was measured by the difference in LC3-2 levels in the presence versus the absence of Bafilomycin A1 inhibitor for each cell sample; (**C**) Autophagy flux was also evaluated in RCC G0 versus RCC null intact cells using the Tandem Sensor RFP-GFP-LC3B Kit. Cells were grown in normal media (0 h) or under starvation conditions for two hours without or with Bafilomycin A1 inhibitor. Cells were stained using the Tandem Sensor RFP-GFP-LC3B reagent and examined using the confocal LSM 880 upright fluorescent microscope. The red and green dots were quantified using the Imaris Image Analysis Software, and the ratio of green to red dots was monitored. Autophagy flux is calculated as LC3-II levels with inhibitors minus LC3-II levels without inhibitors; (**D**,**E**) Western blot analysis and (**F**) Tandem Sensor RFP-GFP-LC3B methodology were used to monitor the autophagy level in RCC G0 cells compared with RCC G1 and RCC G2, as described above. Autophagy flux was measured in both methodologies by comparing the difference in LC3-2 levels in the presence versus the absence of Bafilomycin A1 inhibitor. The uncropped Western blots are shown in Figure S6.

EM images demonstrated an increased number of autophagosomic structures in RCC G1 and G2 compared to RCC G0 parental cells. A previous study indicated that autophagosome maturation was disrupted in mice expressing APOL1 variants G1 and G2, compared to APOL1 G0 expressing mice, resulting in reduced autophagic flux [15]. To examine whether a similar phenotype is apparent in our experimental platform, we first monitored the autophagic flux in RCC G0 cells compared to APOL1 null RCC cells (RCC null #527) in vitro. For this purpose, cells were grown either under normal or starvation conditions to induce autophagy. Under these two conditions, cells were treated either with or without Bafilomycin A1. Bafilomycin A1 is an inhibitor of late phase autophagy and prevents the maturation of autophagic vacuoles by inhibiting fusion between autophagosomes and lysosomes. Autophagic flux was monitored by the cleavage of the LC3 autophagosome marker protein into LC3-I and LC3-II, using Western blot analysis (Figure 6A). The amount of LC3-II at a given time point reflects the number of autophagosomes and autophagy-related structures. The autophagy flux is calculated as LC3-II levels with inhibitors minus LC3-II levels without inhibitors. Quantitation of the results indicated that under normal conditions the autophagy flux is enhanced in RCC null cells compared with RCC G0 cells (0 h) (~3.5 fold) (Figure 6B). This tendency is also observed following two hours of starvation, although to a much lesser extent. It is interesting to note that following four hours of starvation, a lower level of induced autophagy and autophagic flux can be observed both in RCC G0 and in RCC null cells, results that reflect adaptation of RCC cancer cells to starvation conditions [35].

Enhanced autophagy flux in RCC null cells can also be observed by monitoring autophagic flux using the Tandem Sensor RFP-GFP-LC3B reagent, which is based on the differential sensitivity of green fluorescence (GFP) and red fluorescence (RFP) to lysosomal acidity. Since GFP is quenched in acidic compartments, whereas RFP is relatively stable and retained even within lysosomes, the ratio between red and green puncta indicates the level of autophagic flux in the cells. Using this tool, RCC G0 and RCC null cells were stained and visualized by fluorescent microscopy. Red and green signals were quantified, and the ratio of green to red dots was determined. This analysis further supported increased autophagic flux levels in RCC null cells compared with RCC G0 cells under normal conditions (~3 fold) (Figure 6C). Taken together, these results suggest that APOL1 G0 inhibits autophagy in RCC cancer cells.

We next compared the autophagic flux in RCC G0 cells to RCC G1 and RCC G2 cells under normal conditions and under autophagy-induction starvation conditions for two and four hours, without or with Bafilomycin A1. Protein samples were separated on an SDS-PAGE transferred into a membrane, and LC3-I and II proteins were detected using anti LC3 antibody (Figure 6D). Quantitation of the results indicated that under normal conditions (0 h) the autophagic flux is elevated both in RCC G1 (~3 fold) and in RCC G2 (~2 fold) as compared with the level of the autophagic flux in RCC G0 (Figure 6E). Enhanced autophagic flux in RCC G1 and G2 is also observed following two hours of starvation (2 h). However, at four hours, while RCC G0 maintained the autophagic flux level at 2 h of starvation, the autophagic flux level in RCC G1 and G2 was strikingly reduced (Figure 6E). These results suggest that under normal conditions and following two hours of starvation, the capacity of APOL1 G1 and G2 RV to inhibit autophagy in RCC cells was clearly reduced compared to APOL1 G0.

Similar results were observed using the Tandem Sensor RFP-GFP-LC3B reagent. Enhanced autophagic flux was observed in RCC G1 and RCC G2 compared with RCC G0 parental cells under normal conditions and following two hours of starvation (Figure 6F). Overall, our results suggest that APOL1 plays a role in inhibiting autophagy in RCC cancer cells, and that this function is disrupted upon knock-out of APOL1 expression or due to expression of the APOL1 G1 and G2 RV.

### 3.11. The Effect of APOL1 RV on the Tumorigenesis Capacity of RCC Cells

**Figure 7:**
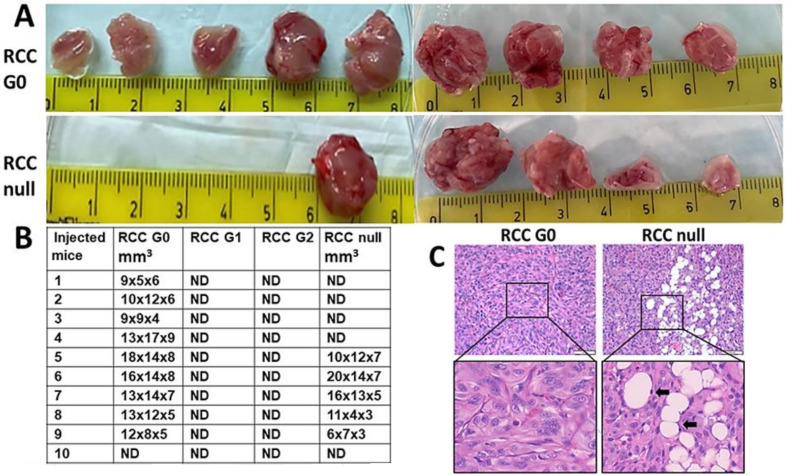
The tumorigenic capacity of APOL1 G0, G1, G2 and null RCC cancer. Cells. Cells were injected intramuscularly (IM) into mice (*n* = 5). (**A**,**B**) Tumors were harvested at 55 days; (**C**) APOL1 G0 RCC-derived tumors demonstrate a dense tissue typical of a highly aggressive epithelioid tumor. APOL1 null RCC-derived tumors exhibit a less dense tissue with multiple foci of differentiation into adipocytes (arrows). Bar = 100 µm for the upper images and 50 µm for the enlarged images.

In previous unpublished studies, we observed a high tumorigenic capacity for RCC 768-0 cancer cells when injected into the hind limb musculature of immunodeficient mice. Hence, we examined whether APOL1 gene editing in these cancer cells affects their tumorigenic capacity in vivo. For this purpose, two independent experiments were carried out. RCC G0 parental cells, RCC APOL1 null, and RCC cells expressing the APOL1 G1 and G2 RV were injected intramuscularly (IM) into mice (*n* = 5). At 55 days, when tumors generated by RCC G0 parental cells reached the size of 1.5 cm^3^, tumors were harvested from all mice, fixed and paraffin embedded for histological analysis. As indicated in Figure 7A,B, 9 out of 10 mice injected with RCC G0 parental cells developed large-size tumors, while only 5 out of 10 mice developed a tumor following injection of RCC APOL1-null cells. None of the mice injected with RCC G1 or RCC G2 developed detectable tumors. Histological examination of tumors generated by RCC G0 cells demonstrated a dense tissue comprised of highly aggressive epithelioid cells with disrupted nuclei, as well as atypical mitotic cells characterized by dispersed chromosomes. In addition, the tumors contain many sinusoidal blood vessels (Figure 7C). The tumor tissue generated following injection of RCC null cells was less dense, with many foci of differentiation into adipocytes. The adipocyte nuclei appeared similar to tumor cell nuclei (Figure 7C). Although no tumors were palpable in mice injected with RCC G1 and RCC G2 cells, the hind-limb musculature tissue was fixed and stained. Indeed, none of the musculature tissues revealed any traces of tumor cells. The failure of RCC G1 and G2 to generate tumors in vivo can be explained by toxicity elicited by the endogenous high expression levels of APOL1 G1 and G2 RV in RCC cells.

## 4. Discussion

The relative risk of renal cell carcinoma associated with chronic kidney disease (CKD) is particularly high among black populations. The estimated incidence of RCC attributable to CKD within black populations accounts for 10%, while it is negligible among Caucasians. However, it is unknown whether the APOL1 risk variants for CKD also contribute to the risk for RCC in black populations [1].

Previously, the mechanisms underlying APOL1 function were investigated mostly in experimental platforms involving ectopic overexpression (conditional or ubiquitous) of APOL1 G0 or APOL1 RV, either in cell lines (HEK293, normal podocytes) or in transgenic mice [15,16,36,37]. Over-expression of APOL1 leads to cytotoxicity via yet unclear mechanisms. The RCC 786-O cell line naturally expresses very high levels of WT *APOL1* (G0), both at the RNA and protein levels (~200 fold compared with normal cells), but interestingly, no signs of APOL1 toxicity are evident. This raised the question of how these cancer cells tolerate high APOL1 levels, and whether they could tolerate high levels of the APOL1 G1 and G2 RV, as well. Additionally, understanding how APOL1 cytotoxicity is abolished in RCC cancer cells could potentially shed light on the mechanism/s by which high levels of APOL1 induce toxicity in normal cells. Here, we describe tissue culture-based studies in RCC 786-O cells that highly express APOL1 G0, G1, or G2 from an endogenous gene copy, achieved by using CRISPR mediated gene editing methodology. Such an approach guarantees that all APOL1 variants are highly expressed in identical genetic backgrounds, thus ensuring that identified phenotypes are attributed specifically to the APOL1 variants. In addition, we used this methodology to knock out the expression of APOL1 and generate APOL1 deficient RCC cells (RCC null). Initial observations indicated slight differences in the proliferation capacity of RCC G0, RCC G1, RCC G2, and RCC null, which suggest that phenotypes related to respiration and energy metabolism are associated with APOL1.

Ultrastructure studies indicated an abnormal mitochondrial phenotype in RCC cells following knock-out or mutations in *APOL1*. This phenotype is characterized by the opening of the mitochondrial cristae (swollen cristae) in RCC null, RCC G1, and RCC G2, as opposed to clear cristae observed in RCC G0 parental cells [38]. Mitochondrial swollen cristae were previously observed in normal podocytes extracted from individuals carrying APOL1 G1 and G2 alleles [20], indicating that in RCC G1 and G2, this phenotype can be unequivocally attributed to the editing of APOL1 and not to an indirect consequence of the CRISPR methodology. These findings suggest a major role for APOL1 in maintaining and controlling the integrity and functionality of the mitochondria in RCC cancer cells and indicate that APOL1 RV may act through a loss of function needed for tumorigenic activity. In addition, we found that the ultrastructure phenotype of RCC G2 and RCC null exhibits short mitochondria containing swelling cristae. On the other hand, RCC G1 exhibits elongated mitochondria with swollen cristae, suggesting a fusion process in RCC G1 cells that can explain the higher proliferation rate and OCR values in this clone compared with RCC null and RCC G2. The differences in mitochondria lengths observed in RCC cells that express APOL1 G1 and G2 and in RCC null cells can be explained by a differential rate of mitochondrial fission and fusion in response to APOL1-related changes in cell metabolism. Mitochondria fusion can occur due to stress that drives the cells to extensively utilize oxidative phosphorylation as a source for ATP production, specifically by withdrawing glucose as a carbon source for cell metabolism [33]. Fusion can also compensate for mitochondrial defects by complementing respiratory and ATP production functions [33].

High levels of cellular stress can additionally lead to apoptosis, which can also culminate in excessive mitochondrial fission [33]. Nevertheless, we could not detect a significant difference in the mRNA levels of the mitochondrial fission and fusion genes FIS1 and MFN1, as was observed in normal podocytes displaying a similar phenotype (data not shown) [20]. Overall, the modulation of the mitochondrial structure and the abnormal phenotypic appearance of the mitochondrial cristae point to physiological stress induced in RCC cells following the alterations we generated in the APOL1 gene, and suggest that abnormal mitochondrial metabolism levels are linked to APOL1 risk variants or lack of APOL1 expression.

Since RCC are high energy demanding cancer cells, as indicated by their proliferation rates in vitro and in vivo, it was important to examine the mitochondrial function of APOL1 variants by an analysis that directly measures mitochondrial respiration. Mitochondrial dysfunction is difficult to study in a high glucose medium as rapidly proliferating cancer cells are highly glycolytic in such medium [28]. The production of pyruvate via glycolytic metabolism of glucose yields two net ATP molecules, whereas the production of pyruvate via glycolytic metabolism of galactose yields no net ATP molecules. Therefore, galactose-dependent metabolism forces cells to rely on mitochondrial respiration via oxidative phosphorylation (OXPHOS) for energy production [29]. Therefore, to reliably measure proliferation rates and mitochondrial metabolism, we substituted galactose for glucose in the growth medium [29]. Indeed, RCC G0, G1, G2, and RCC null exhibit similar proliferation rates in vitro when grown in high glucose media, as they can compensate for mitochondrial dysfunction by utilizing glycolysis for ATP production. In contrast, G0 and G1 cells reached higher confluence when grown in galactose supplemented medium (as the sole carbohydrate source) compared to G2 and null cells. The differences in the mitochondrial structure and in the proliferation rates observed in RCC cells as a result of eliminating or editing APOL1 were integrated with bioenergetics values obtained by the metabolic flux technology of the Seahorse bioanalyzer. This analysis supports the notion that the genetic manipulation of APOL1 in RCC cancer cells leads to an alteration in mitochondrial metabolism, which in turn influences downstream gene expression, signal transduction, and the tumorigenic capacity of the cells [39]. The most significant bioenergetic consequence of APOL1 manipulation was the loss of ATP-coupled respiration in APOL1 null cells, and to a lesser extent in G2 isoform expressing cells.

In high energy demanding cells, mitochondria cristae packaging is tightly regulated to ensure proper respiratory processes [40]. The swollen mitochondrial cristae in RCC G1, G2, and in RCC null are consistent with a deregulated process wherein mitochondrial inner membrane unfolding leads to rupture of the outer membrane and to the release of pro-apoptotic molecules that eventuate in cell death [41]. It has been demonstrated that swollen mitochondria cristae are associated with increased calcium concentrations and can disrupt mitochondrial function [41,42]. A recent study indicates that while all three APOL1 variants traffic to the plasma membrane (PM), only G1 and G2 RV, but not G0, have an enhanced capacity to form cation channels that enable Na^+^, K^+^, and Ca^2+^ conductance [23]. Thereby, we postulate that APOL1 G1 and G2 channel activity can augment cytotoxic influx of Na^+^ and Ca^2+^ that leads to uncontrolled gene expression, deregulated signaling pathways, cell swelling, and eventually to APOL1 associated cell death [23]. APOL1 G1 and G2 cation channel-mediated cytotoxicity were also demonstrated when overexpressed in Human Embryonic Kidney 293 (HEK293) cells [36]. These observations suggest that intracellular changes in Ca^2+^ concentration additionally affect intra mitochondrial Ca^2+^, leading to mitochondrial cristae swelling, disturbed metabolism, and eventually to cell cytotoxicity. In any case, the rise in cytosolic Ca^2^+ needs to be considered and measured as part of future studies to unravel the serial steps leading to observed mitochondrial swelling. The fact that swelling mitochondria and disturbed metabolism are also observed in RCC APOL1-null cells implies that APOL1 RV cytotoxicity is mediated via additional unknown mechanisms linked to mitochondria function. Yet, we demonstrated that re-expression of APOL1 G0 in RCC null cells could significantly rescue the swelling cristae phenotype in these cells and improve their proliferation capacity, suggesting an APOL1 specific loss of function. The rescue of the mitochondrial phenotype by APOL1 G0 additionally supports the conclusion that the mitochondrial phenotype apparent in RCC null cells is directly associated with APOL1 loss of function and not an off-target effect of the editing procedure [43].

Autophagy, which was also proposed as a mechanism responsible for APOL1 RV cytotoxicity, is associated with increasing levels of APOL1 expression in normal and cancer cells [13,14,15]. Nevertheless, autophagy as a mechanism that mediates APOL1 associated cytotoxicity appears cell-dependent [13,18]. As high energy demanding cells, RCC cancer cells require high autophagy activity to remove damaged organelles via the lysosomal pathway and recycle them for cell growth, energy production, and nutrient consumption during starvation or stress [44]. In the current study, we observed a significant elevation in autophagic flux in APOL1-null RCC cells, as well as in RCC cells that express APOL1 G1 and G2 RV. Hence, we suggest that these RCC cells, which exhibit mitochondrial dysfunction at both the morphological and functional levels, induce autophagy (and mitophagy) to remove damaged mitochondria. A previous study suggested that changes in Ca^+2^ can lead to mitochondria swelling cristae and increased autophagy in the cells [42]. However, elevation in autophagic flux both in the absence of APOL1 expression and in the presence of APOL1 RV might indicate that APOL1 RV-induced attenuation in respiration and growth capacity is independent of autophagy. These observations agree with a previous observation that autophagy is a response to APOL1 RV cytotoxicity rather than a mediator [36]. The fact that elimination of APOL1 expression and the expression of G1 and G2 RV elicit similar effects in RCC cells might suggest that APOL1 loss of function in the cells can be mediated by more than one pathway, which eventually lead to cell death.

Proliferation and autophagy are considered two major hallmarks of cancer [34]. Our findings that elimination or alteration of APOL1 in RCC leads to attenuated cell growth and metabolism and increased autophagy prompted us to examine whether these changes play a role in the tumorigenic capacity. Using in vivo tumor generation as the gold standard for tumorigenic potential, we observed that RCC cancer cells which express the APOL1 G1 and G2 RV fail to generate tumors in mice. Such an absolute elimination of RCC cell tumorigenic potential is puzzling given that RCC null cells succeeded in generating tumors, albeit at lower rates than G0 RCC cells. We postulate that the extreme elimination of the tumorigenic potential might result from APOL1 G1 and G2 cytotoxicity, which arises under conditions specific to in vivo growth, such as relatively low oxygen levels. However, under in vitro growth conditions that include a high glucose containing medium, APOL1 G1 and G2 are less toxic to RCC cells. The reduced in vivo tumorigenic potential of RCC G1 and G2 predicts that human carriers of these variants may be relatively protected from RCC and suggests that the high incidence of RCC in the black population is unrelated to the APOL1 RV for CKD. Future studies are necessary to determine the association of APOL1 G1 and G2 RV with kidney cancer in Sub-Saharan and African-American populations compared to populations of European ancestry.

The RCC G0 parental cells generated aggressive tumors with a condensed tissue appearance typical to RCC. Interestingly, the tumors generated by RCC null cells displayed a less condensed tumor tissue, with many foci of differentiation into adipocytes, carrying typical RCC cell nuclei. This observation might indicate an epithelial to mesenchymal transition (EMT) process occurring in RCC null cells growing in murine musculature and implies that APOL1 G0 inhibits EMT. Future studies related to such a role executed by APOL1 may shed light on the regulation of EMT in kidney cancer and be useful for devising therapeutic avenues for kidney-related cancers.

## 5. Conclusions

We recognize the potential limitations of the current study, particularly the possible influence of clonal variation affecting the cell lines derived from single cells following genome editing. We have attempted to substantially address this limitation, using the reconstitution studies in which APOL1-null cells were “repaired” in their growth and mitochondrial structural and functional phenotypes by reintroducing the G0-APOL1. We recognize that further studies with bulk populations representing average responses for each somatic genotypic state of interest for APOL1 or numerous single cell derived clones might be needed as a follow-up.

However, besides representing the first study to our knowledge in which differences in cellular behavior are observed for endogenous APOL1 variants on the same isogenic background, the current study shows a complex cell survival/injury duality. On the one hand, the expression of endogenous APOL1 G0 is needed for RCC cell growth and tumorigenicity, even though APOL1 is considered dispensable for normal kidney cell function and known human health, except under pathogen attack. On the other hand, the kidney disease risk variants do not replace APOL1 G0 in this regard but rather demonstrate the well-known cellular toxicity that also prevents RCC cell growth and hence tumorigenic potential. The need for APOL1 variant genetic epidemiology studies of RCC in African ancestry populations is now even more strongly motivated.

## Supplementary Materials

The following are available online at https://www.mdpi.com/article/10.3390/cancers14030733/s1, Figure S1: Fluorescent in situ Hybridization (FISH) analysis on (A) RCC nuclei and (B) metaphase chromosomes indicated three copies of APOL1 in these cells, Figure S2: Proliferation capacity of RCC G0, RCC G1, RCC G2, and RCC null cells, Figure S3: Uncropped Western blots for Figure 1, Figure S4: Uncropped Western blots for Figure 3, Figure S5: Uncropped Western blots for Figure 5, Figure S6: Uncropped Western blots for Figure 6.
