# Supplementary material for: Comparative Analysis of the APOL1 Variants in the Genetic Landscape of Renal Carcinoma Cells
Source: Cancers (Basel). 2022 Jan 30;14(3):733. doi: 10.3390/cancers14030733 (PMC8833631; doi:10.3390/cancers14030733)
Supplement: Supplementary file 1 [file cancers-14-00733-s001.zip › cancers-1529655-supplementary.pdf]

cancers

MDPI

Supplementary Materials: Comparative Analysis of the APOL1 Variants in the Genetic Landscape of Renal Carcinoma Cells

Maty Tzukerman, Yeela Shamai, Ifat Abramovich, Eyal Gottlieb, Sara Selig and Karl Skorecki

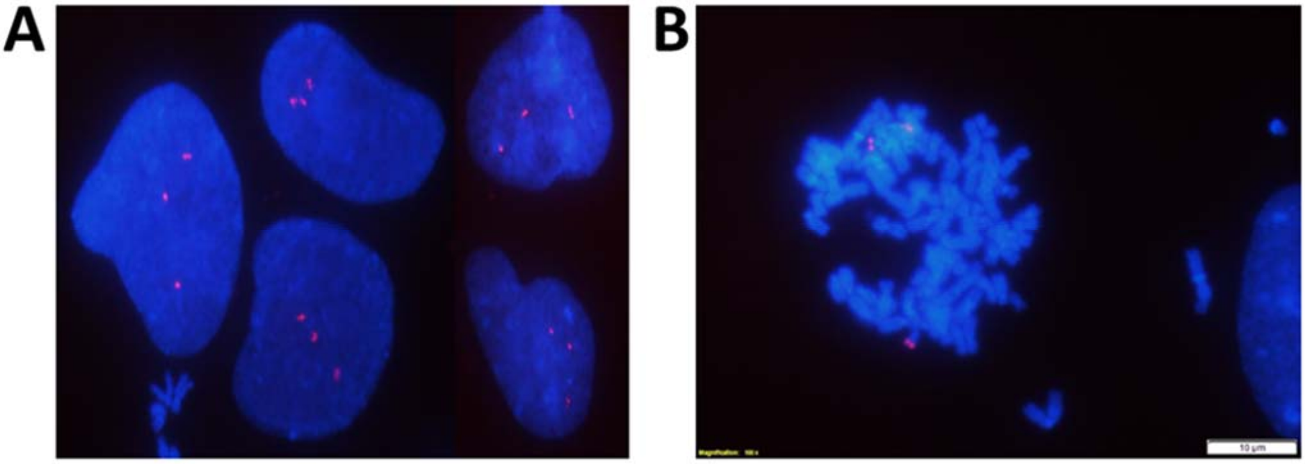

Figure S1. Fluorescent in situ Hybridization (FISH) analysis on (**A**) RCC nuclei and (**B**) metaphase chromosomes indicated three copies of *APOL1* in these cells. RCC cells were treated with colcemid, harvested by trypsinization, treated with hypotonic solution and fixed with methanol/acetic acid (3:1). Cells were dropped on slides and hybridized by a standard FISH protocol, to a probe generated from an *APOL1*-region BAC clone (RP1-6802). Probe was labelled with dUTP-digoxigenin. Hybridization was detected with anti-Dig-Rhodamine. DNA was stained with DAPI. Nuclei and chromosomes were visualized on a BX50 microscope (Olympus). Images were captured with an Olympus DP70 camera controlled by DP controller software (Olympus). Bar = 10 μm.

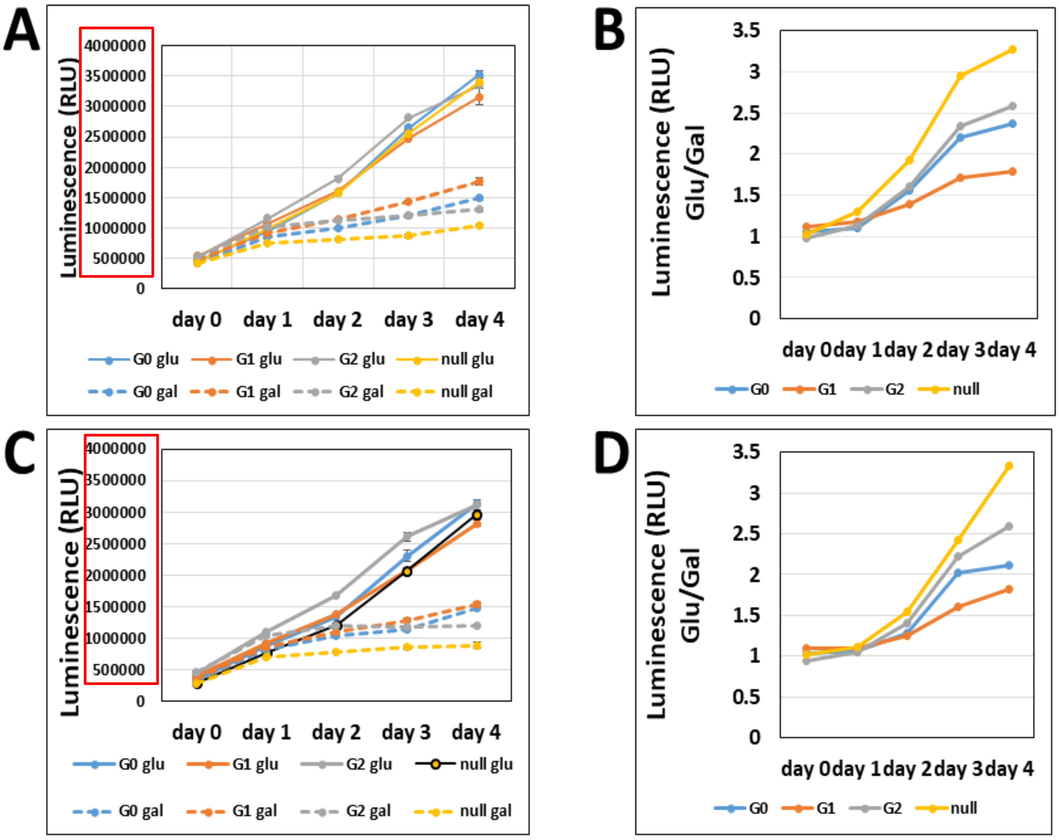

Figure S2. Proliferation capacity of RCC G0, RCC G1, RCC G2 and RCC null cells. Cells were seeded in an opaque-walled 96 well plates in culture medium that contains glucose or galactose as a carbohydrate source of energy, in quadruplicates. Cells were measured for proliferation capacity at days 0, 1, 2, 3, and 4, using the CellTiter-Glo® luminescent cell viability assay. Luminescence values presented as relative light units (RLU) correspond to the number of cells in each examined day, and the proliferation rate is presented for each type of cell (**A** and **C** represent two independent experiments). The ratio of proliferation rate on glucose to the proliferation rate on galactose, for each type of RCC cells in each experiment, are presented in **B** and **D**.

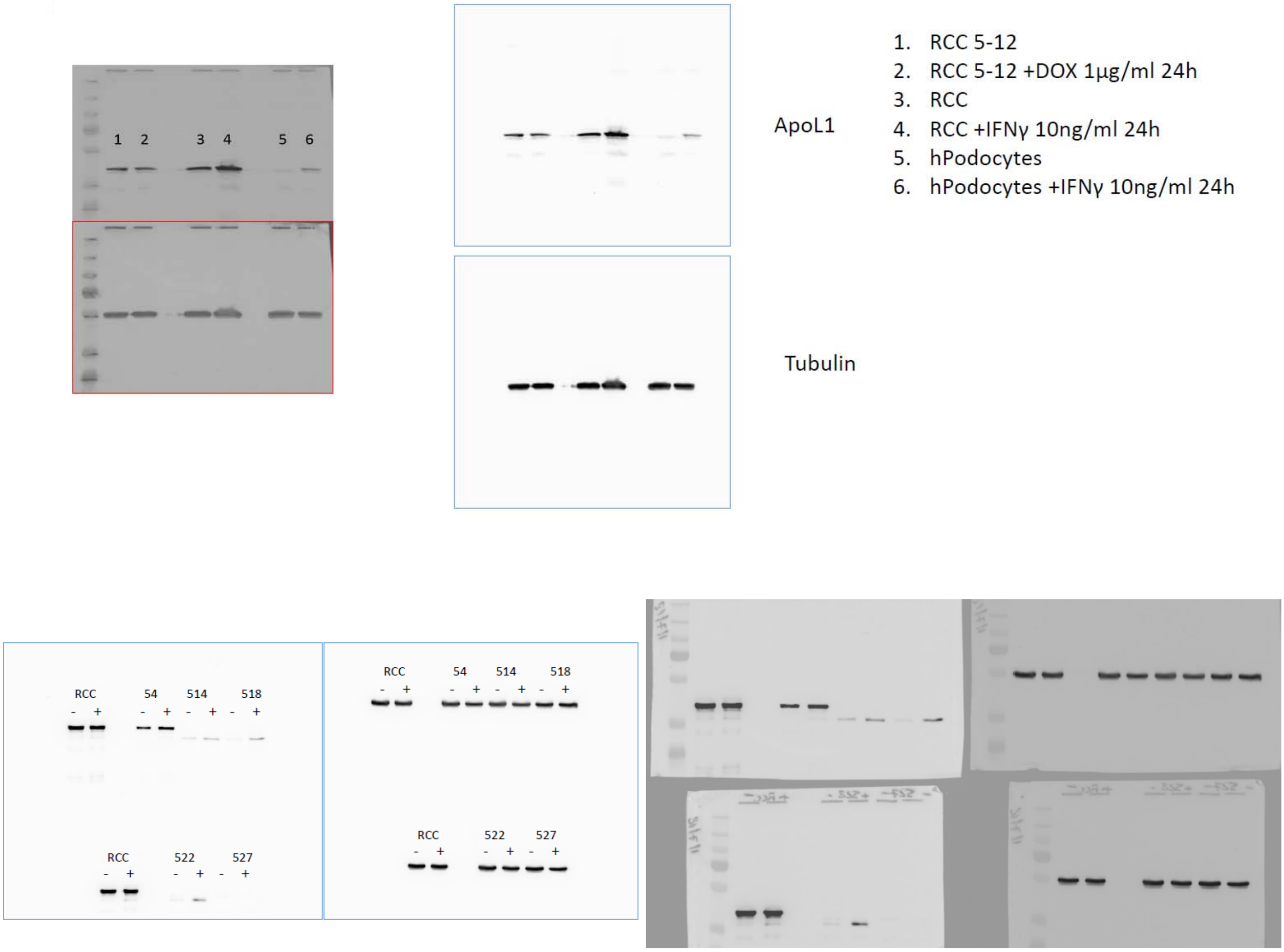

Figure S3. Uncropped Western blots for Figure 1.

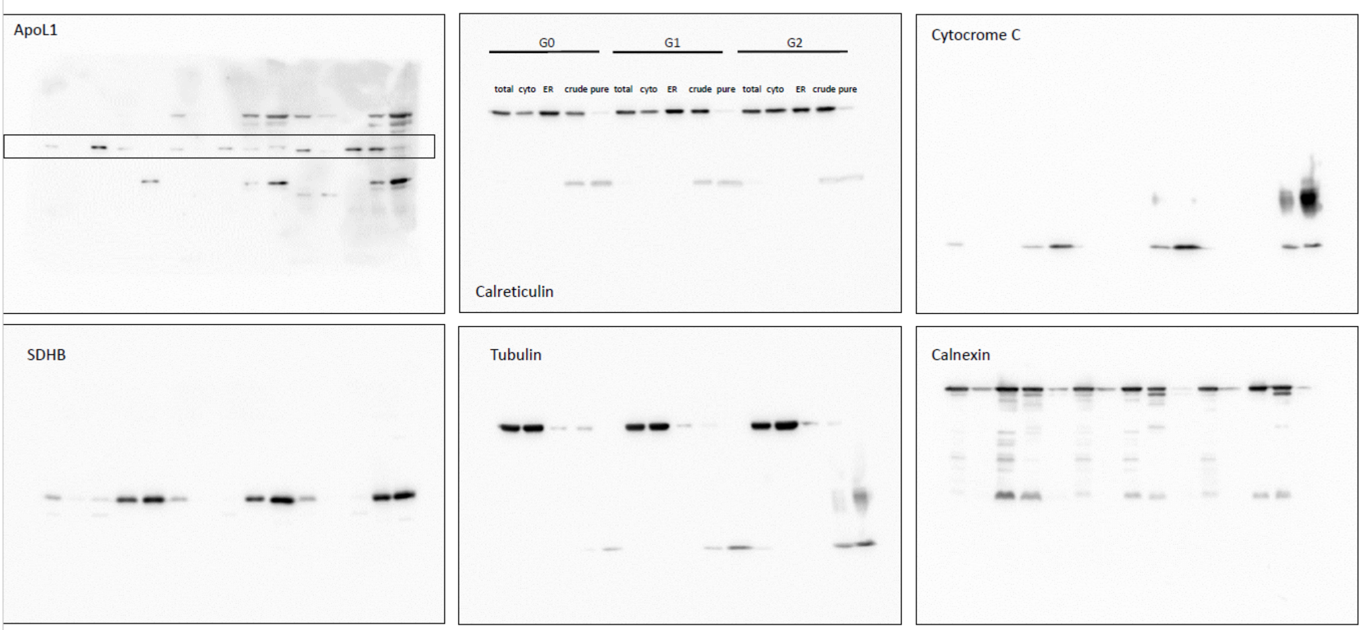

Figure S4. Uncropped Western blots for Figure 3.

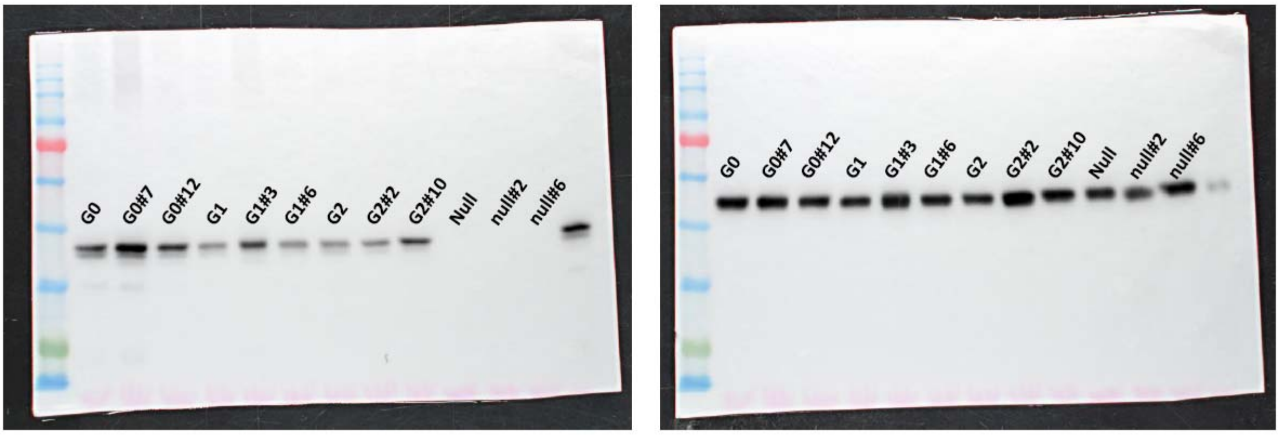

Figure S5. Uncropped Western blots for Figure 5.

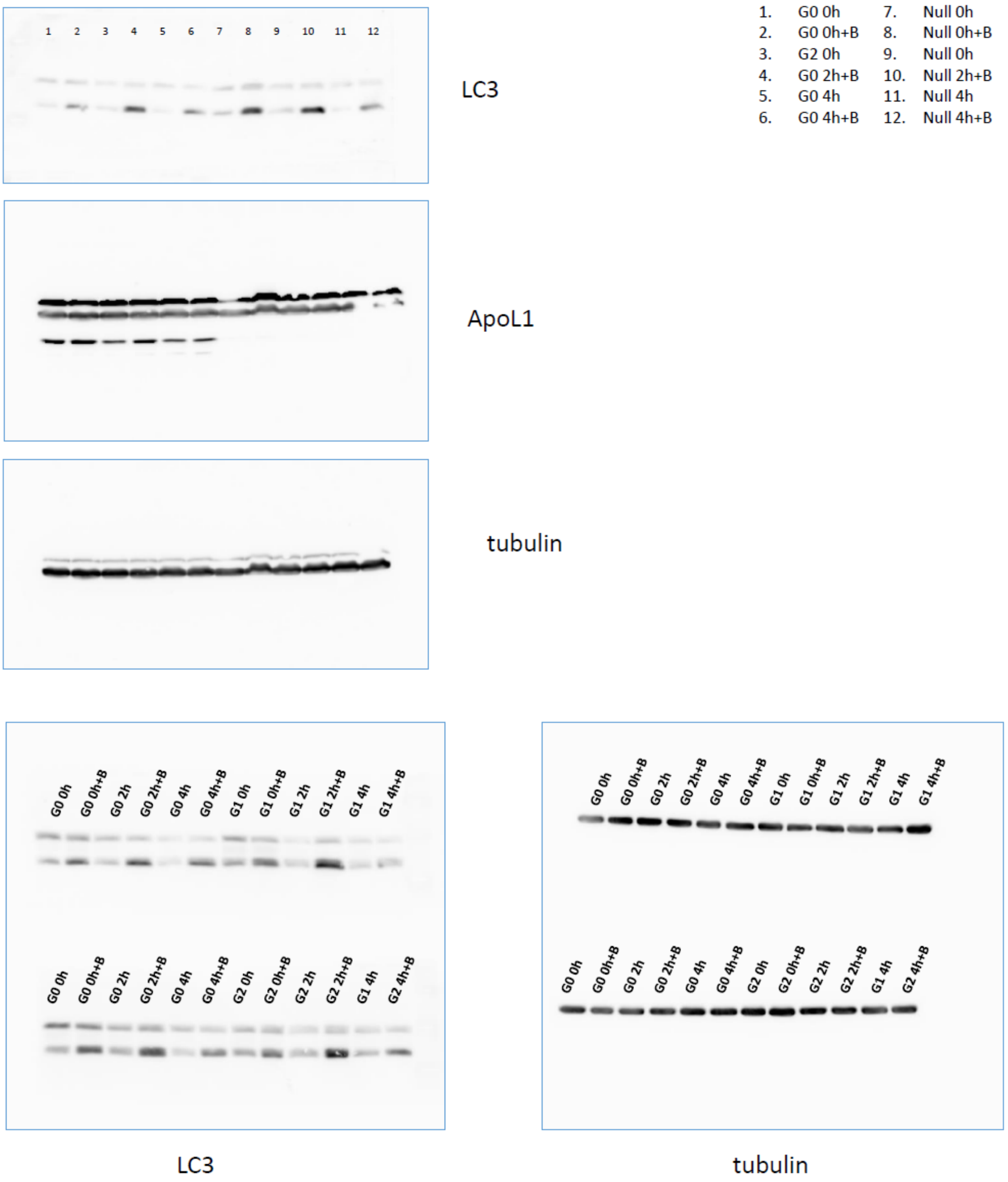

Figure S6. Uncropped Western blots for Figure 6.
